# Immune Parameters in Chickens Treated with Antibiotics and Probiotics during Early Life

**DOI:** 10.3390/ani12091133

**Published:** 2022-04-28

**Authors:** Jan Jankowski, Bartłomiej Tykałowski, Anna Stępniowska, Paweł Konieczka, Andrzej Koncicki, Paulius Matusevičius, Katarzyna Ognik

**Affiliations:** 1 Department of Poultry Science and Apiculture, Faculty of Animal Bioengineering, University of Warmia and Mazury in Olsztyn, Oczapowskiego 5, 10-719 Olsztyn, Poland; janj@uwm.edu.pl (J.J.); pawel.konieczka@uwm.edu.pl (P.K.); 2 Department of Poultry Diseases, Faculty of Veterinary Medicine, University of Warmia and Mazury, Oczapowskiego 13, 10-719 Olsztyn, Poland; bartlomiej.tykalowski@uwm.edu.pl (B.T.); andrzej.koncicki@uwm.edu.pl (A.K.); 3 Department of Biochemistry and Toxicology, Faculty of Animal Science and Bioeconomy, University of Life Sciences in Lublin, Akademicka 13, 20-950 Lublin, Poland; anna.stepniowska@up.lublin.pl; 4 Department of Animal Nutrition, Lithuanian University of Health Sciences, Kaunas, Tilzes 18, LT-47181 Kaunas, Lithuania; paulius.matusevicius@lsmuni.lt

**Keywords:** chicken, antibiotic, probiotic, immune response

## Abstract

**Simple Summary:**

The effect of using antibiotics in animal production is resulting in an increase in drug resistance among bacteria, often to multiple substances with different mechanisms of action. In the future, this could lead to an increase in mortality in human and animal populations. In consequence, there is enormous and fully justified public pressure to limit the amount of antibiotics used in livestock production. The study aimed to compare the effect of administration of antibiotics or probiotics on chickens in their first week of life, on selected parameters of humoral and cellular immunity, and on the bursa of Fabricius and spleen indices. Administration of the antibiotic enrofloxacin or a probiotic containing *Enterococcus faecium* and *Bacillus amyloliquefaciens* strains to chickens in their first week of life exerts pronounced immunomodulatory effects on humoral and cellular defence mechanisms in these birds. Early administration of a probiotic has a positive effect on the immune system, however, early administration of enrofloxacin can pose a risk of suppression of humoral immunity of the chickens.

**Abstract:**

The aim of the study was to compare the effect of the administration of antibiotics or probiotics on chickens in their first week of life, on selected parameters of humoral and cellular immunity, and on the bursa of Fabricius and spleen indices. The experiment was carried out on 90 one-day-old male broilers. The control group received no additive in the drinking water; the group GP received a probiotic providing *Enterococcus faecium* and *Bacillus amyloliquefaciens*; and the group GA received 10% enrofloxacin in the drinking water on the first five days of life. Administration of the antibiotic enrofloxacin or a probiotic containing *E. faecium* and *B. amyloliquefaciens* strains to chickens in their first week of life exerts pronounced immunomodulatory effects on humoral and cellular defense mechanisms in these birds. The changes in the subpopulations of B and T cells immediately following early administration of enrofloxacin or the probiotic were not observed at the age of 35 days. Early administration of enrofloxacin can pose a risk of suppression of humoral immunity, as indicated by the significant decrease in the total IgY concentration in the plasma of the chickens.

## 1. Introduction

About 70% of the global supply of antibiotics and chemotherapeutics is consumed in animal production, and a further increase is expected by 2030 as a result of the predicted increase in the number of farm animals. This is due to the rising demand for meat in the continually growing human population, especially in developing countries [1]. The effect of the use of antibiotics in animal production is an increase in drug resistance among bacteria, often to multiple substances with different mechanisms of action [1,2]. In the future, this could lead to an increase in mortality in human and animal populations [2]. In consequence, there is enormous and fully justified public pressure to limit the amount of antibiotics used in livestock production. 

For many decades poultry production has been the fastest-growing sector of animal production. Contemporary, fast-growing broiler chickens are highly sensitive to adverse environmental conditions associated with intensive production, which lead to immune suppression and infection even by pathogens with low virulence [1]. The practice among many poultry producers is the metaphylactic administration of antibiotics to chickens in the first few days of life and, in some cases, also at the time of first vaccination [3,4]. This can negatively affect the defense mechanisms of birds, determined by the functioning of the major immune system organs, which are the site of lymphocyte differentiation and development [5]. 

One of the drugs most commonly used in poultry production is enrofloxacin, a fluoroquinolone with a broad spectrum of activity against Gram-positive and Gram-negative bacteria [3,6]. There are reports indicating that although enrofloxacin inhibits humoral immune mechanisms [7], it can benefit the cellular immune response in chickens [6]. Detailed studies on the effect of fluoroquinolones on the functioning of immunocompetent cells in vitro and in vivo showed that they interfered significantly with the expression of many cytokines [8]. Studies have been conducted for many years on substances positively affecting the gut microbiota of chickens while stimulating their defense mechanisms, thereby reducing the need for the preventive and therapeutic use of antibiotics. Probiotics are unquestionably foremost among these substances [9,10,11,12,13,14]. According to FAO/WHO definition: probiotics are live microorganisms that, when administered in adequate amounts, confer a health benefit on the host [15]. Many poultry producers have replaced preventive administration of antibiotics in the first few days of life with supplementation with probiotics [16,17]. Some producers apply probiotic bacteria to bedding material in order to colonize it with beneficial microbes and displace pathogenic microbes in the environment of poultry [18]. According to Cheng et al. [19], probiotics help to maintain the physiological microbiota of the digestive tract, modulate the host immune response, and lower the risk of infection with pathogenic bacteria, which positively affects production outcomes. Studies in broiler chickens show that dietary supplementation with probiotics, such as those based on *Bacillus*, *Lactobacillus*, and *Clostridium*, can improve humoral immunity [20,21,22] and inhibit the multiplication of pathogenic bacteria in the digestive tract [23]. 

The aim of the study was to compare the effect of administration of an antibiotic or probiotic to chickens in their first week of life on selected parameters of humoral and cellular immunity and on the bursa of Fabricius and spleen indices.

## 2. Materials and Methods

### 2.1. Chicken Experiment

The chicken experiment is described in an earlier publication by Ognik et al. [12]. The experiment was carried out on 90 one-day-old male broilers of the Ross 308 strain with an initial body weight of 42.8 ± 0.90 g, purchased from a local commercial hatchery. The birds were randomly assigned to three dietary treatments with 30 birds in each treatment and placed in pens on litter (wood shavings). The housing conditions were in accordance with standard management practices for commercial chicken houses. The birds were fed commercial starter and grower diets formulated to meet or exceed their nutritional requirements in accordance with their age [24]. Feed composition was presented in our previous publication [12]. The three treatments were as follows: the control group received no additive in the drinking water (GC); the second group (GP) received a probiotic preparation in the drinking water on the first five days of life, providing *Enterococcus faecium* strain 4a1713 at 1.0 × 10^7^ CFU/L water and *Bacillus amyloliquefaciens* 4b1822 at 1.0 × 10^7^ CFU/L water, according to the manufacturer (BioPoint. Poland); and the third group (GA) received 10% enrofloxacin in the amount of 0.5 mL/L (Scanoflox 10% Oral, Lavet Pharmaceuticals Ltd., Budapest, Hungary) in the drinking water on the first five days of life. The drinking water in each group was changed daily during the five-day period, and a fresh preparation of either the probiotic supplement or the antibiotic was provided. The birds were reared until the age of 35 days, and their body weight and feed intake were measured. 

### 2.2. Sampling Procedures

At 6 and 35 days of age, 10 chickens from each group were slaughtered by decapitation following electrical stunning. Following decapitation, samples of the blood, spleen, and bursa of Fabricius were excised and weighed. The spleen index and bursa of Fabricius index were calculated as the ratio of spleen or bursa of Fabricius weight to body weight. Blood for analysis was collected into test tubes with heparin (for biochemical analyses) or with EDTAK2 (for cytometric analyses). Next, the blood samples for biochemical analyses were centrifuged at 3000× *g* for 10 min, and the plasma was collected for further analysis. In the blood plasma levels of immunoglobulins A and Y (IgA and IgE), interleukin-6 (IL-6), interleukin-2 (IL-2), tumor necrosis factor α (TNF-α), C reactive protein (CRT), and activity of ceruloplasmin (Cp) were determined using commercial measurement ELISA kits (FineTest, Wuhan Fine Biotech Co., Ltd., Wuhan, China). Albumin level (ALB) was determined using biochemical kits (Cormay, Warsaw, Poland).

### 2.3. Isolation of Mononuclear Cells and Flow Cytometry

**Figure 1:**
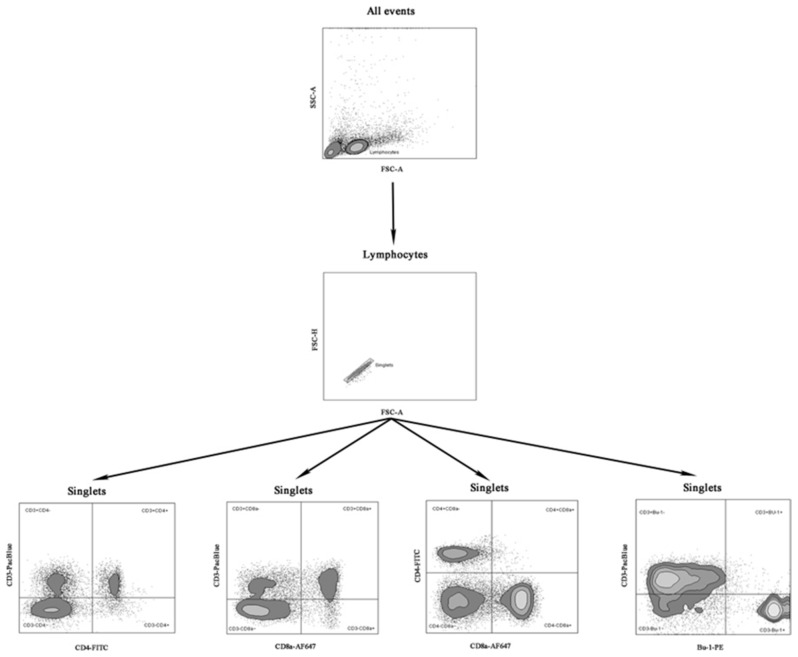
Gating strategy for extracellular staining for CD3+CD4+, CD3+CD8a+, CD4+CD8a+ T and CD3-Bu-1+ B cells in samples of the examined chickens. Abbreviations: FSC-A: forward scatter area, FSC-H: forward scatter height SSC-A: side scatter area.

Mononuclear cells from the blood and spleens were isolated according to a previously described procedure [25]. The cells were counted, and their viability was evaluated using the Vi-Cell XR cell counter (Beckman Coulter, Brea, CA, USA). Viable mononuclear cells (1 × 106) were stained with Pacific Blue conjugated Mouse Anti-Chicken CD3-PACBLU clone CT-3, fluorescein-conjugated Mouse Anti-Chicken CD4-FITC clone CT-4, Alexa Fluor 647 conjugated Mouse Anti-Chicken CD8a-AF647 clone CT-8, and phycoerythrin-conjugated Mouse Anti-Chicken Bu-1-PE clone AV-20 (Southern Biotech, Birmingham, AL, USA). Data were acquired using a FACSCanto II digital flow cytometer (BD, USA) in the FACSDiva 8.0 environment (BD, USA). The 50,000 events were recorded for each sample at an average flow rate of approximately 1500 events per second. The immunophenotype and percentages of subpopulations of T CD3+CD4+, CD3+CD8a+ lymphocytes, double-positive cells (CD4+CD8a+), and B cells (Bu-1+) were analyzed using FlowJo V10 software (BD, USA). Fluorescence minus one (FMO) controls for FITC, PE, Pacific Blue, and Alexa Fluor 647 fluorochromes were used to determine the cut-off point between background fluorescence and positive populations. The cytometer setup and tracking beads (CST, BD, USA) were used to initialize photomultiplier tubes settings. Unstained and single-stained control cells for each fluorochrome were prepared and used to set up flow cytometry compensation. Figure 1 presents the gating strategy using an example of a spleen sample.

### 2.4. Ethical Statement

All procedures involved in handling the birds were performed by qualified veterinarians. No action involving pain or suffering was practiced, and all of the analyses were performed on samples collected post-mortem. The protocol for this study and the number of chickens used in this study were consistent with the regulations of the Local Committee for Experimentation on Animals (Olsztyn, Poland) and were performed in accordance with the principles of the European Union Directive 2010/63/EU for animal experiments and Polish Law on Animal Protection. 

### 2.5. Statistical Analysis

Data are presented as the mean ± standard error of the mean (SEM) (*n* = 10 for each group). Differences between groups were determined by one-way ANOVA using Tukey’s HSD test. The significance level was set at *p* < 0.05. All calculations were performed using the GLM procedure of STATISTICA software version 12.

## 3. Results

Growth performance results were presented in our previous paper [12]. At 35 days of age, chickens from group GP had a higher body weight than chickens from group GC or GA. In comparison with group GC, spleen weight, the bursa of Fabricius weight, and the bursa of Fabricius index at 6 days of age in groups GP and GA was higher (*p* = 0.047; *p* = 0.036; *p* = 0.004, respectively), whereas at 35 days of age no statistical differences were noted between any groups of chickens (Table 1). 

In the spleen of 6-day-old chickens from the groups receiving a probiotic or an antibiotic, the percentage of CD3+CD4+ T cells was lower (*p* = 0.001), but that of CD3-Bu-1+ B cells was higher (*p* = 0.001) than in chickens from the GC treatment (Table 2).

The percentage of CD3-Bu-1+ B cells in the blood of 6-day-old chickens from groups GP and GA was also higher (*p* = 0.007) than in the control group (Table 3). At 35 days of age, plasma Cp and CRP levels in chickens from group GA were lower (*p* < 0.001, both) than in the control group. The plasma level of IgY in the chickens from the GA treatment was lower at both 6 and 35 days of age (*p* = 0.014; *p* < 0.001, respectively) than in the GC treatment. Compared to the control group, 6- and 35-day-old chickens that had received enrofloxacin had higher plasma levels of IL-2 (*p* < 0.001, *p* = 0.023, respectively). The plasma level of IL-6 in the 6-day old chickens from the group receiving the probiotic was lower (*p* = 0.002) than in the control group. The 6- and 35-day-old chickens in group GP had lower plasma activity of Cp (*p* = 0.02 and *p* < 0.001, respectively) and CRP (*p* < 0.001, both; Table 4).

## 4. Discussion

The bursa of Fabricius, spleen, and thymus are organs with an important role in cellular and humoral immunity in birds. The bursa of Fabricius is the main lymphoid organ in which proliferation and differentiation of B cells takes place [26]. According to many authors, the value of the bursa of Fabricius index, i.e., the ratio of its weight to body weight, is a measure of the effect of various infectious and non-infectious factors on the functioning of the immune system of birds [5,27,28]. Yin et al. [27] and Heckert et al. [28] state that immunosuppressive factors inhibit the growth of the bursa of Fabricius. Ellakany et al. [5], in a study in chickens receiving enrofloxacin on the first five days of life at therapeutic doses or at 10 times that level, the antibiotic was not found to affect the bursa of Fabricius index. In the present study, early administration of enrofloxacin or a probiotic containing *Enterococcus faecium* and *Bacillus amyloliquefaciens* led to an increase in the bursa of Fabricius index in the chickens. The results indicate that both the probiotic used in the experiment and enrofloxacin had a beneficial effect on the size of this organ at 6 days of age. According to many authors, the development and maturation of immune system organs is more effective in healthy birds than in sick ones, and their size may be indicative of normal functioning [29,30,31]. Zhang et al. [32] reported an increase in the weight of the bursa of Fabricius in chickens receiving probiotics containing *Lactobacillus casei*, *Lactobacillus acidophilus*, or *Bifidobacterium* in their drinking water. In contrast, Fathi et al. [22] found that administration of 200 or 400 mg/kg of a probiotic containing 4 × 109 CFU/g *Bacillus subtilis* to broiler chickens had no effect on the weight of the bursa of Fabricius.

A study by Chrząstek et al. [4] showed that early administration of antibiotics (including enrofloxacin) to chickens at therapeutic did not cause disturbances in the microscopic structure of the bursa of Fabricius, but does cause a significant decrease in the percentage of Bu-1+ B cells in this organ. Previously, Ellakany et al. [5] established that enrofloxacin at 10 times the therapeutic dose significantly reduced the number of B cells in the peripheral blood, although it did not affect the weight of the bursa of Fabricius. In the present study, the increased percentage of CD3-Bu-1+ cells in both the spleen and blood of 6-day-old chickens receiving enrofloxacin for the first 5 days of life indicates that the antibiotic did not have a suppressive effect on this subpopulation of lymphocytes. The significant increase in the percentage of B lymphocytes (CD3-Bu-1+) in the blood and spleen of 6-day-old chickens is probably linked to the high plasma level of IL-2 noted in the blood at that time, as it stimulates proliferation of both CD3+CD8+ cells and B cells. In the present study, the percentage of CD3+CD8a+ T cells in the blood of 6-day-old chickens was highest in the group receiving enrofloxacin. The data correspond with the findings of many studies on the mechanism of the immunomodulatory effect of quinolones, presented by Dalhoff and Shalit [8]. In poultry species, interleukin 2 affects lymphocyte proliferation, activation of NK cells, and clearance of intracellular pathogens [33,34]. In a study by Wisselink et al. [35], administration of enrofloxacin to chickens increased mRNA expression of genes encoding IL-2. The presence of certain antimicrobial drugs may cause modifications in phagocyte and lymphocyte functions and reduce the production of pro-inflammatory cytokines and oxidation processes (respiratory burst) [36]. This, in turn, leads to the suppression of chronic inflammation in the body in in vivo conditions. These factors, especially at subinhibitory concentrations (below the MIC value), can also fundamentally alter the morphology, metabolism and/or virulence of pathogens, rendering them susceptible to the effects of immunocompetent cells [36,37].

Our study also showed an increase in the CD3-Bu-1+ cell subpopulation in both the blood and the spleen of 6-day-old chickens receiving a probiotic containing *Enterococcus faecium* and *Bacillus amyloliquefaciens*. While neither enrofloxacin nor the probiotic was found to affect the spleen index, there was a significant decrease in the percentage of CD3+CD4+ T cells in the spleen of 6-day-old chickens from groups GP and GA. The significant reduction in the subpopulation of T cells, which was particularly pronounced in group GA is most likely linked to the inhibitory effect of enrofloxacin on the proliferation of these lymphocytes. The obtained data partially correspond to the results obtained by Chrząstek and Wieliczko [3], which showed in their experiment that the administration of enrofloxacin to chickens in the first days of life resulted in a decrease in the percentage of CD4+CD8- T lymphocytes, Bu-1+ B cells and an increase in the percentage of CD4-CD8+ T cells in the spleen of 14-day-old birds. 

However, Williams et al. [38] reported that ciprofloxacin and moxifloxacin (quinolones similar to enrofloxacin) inhibit secretion of IL-4 and IFN-gamma by CD3+CD4+ cells. These cytokines are the main factors determining the direction of defense mechanisms towards type Th1 (IFN-γ) or Th2 (IL-4). Blockage of IL-4 secretion may lead to a significant decrease in antibody synthesis, which is in line with our observations of the blood level of IgY antibodies in chickens receiving enrofloxacin. The level of this immunoglobulin was lowest in chickens receiving enrofloxacin at both 6 and 35 days of age, although the percentage of B cells in the blood and spleen at 6 days of age was the highest in these birds. Perhaps it is related to the inhibitory effect of this antibiotic on the expression of IL-4 in Th2 cells, however, in our experiment, this indicator was not determined. Furthermore, Khalifeh et al. [6] found in their studies that the administration of enrofloxacin to SPF chickens prior to vaccination against Newcastle Disease significantly reduces the serum levels of post-vaccination HI antibodies in these birds. Wisselink et al. [35] drew attention to a decrease in the number of CD4+ and CD8+ cells in the duodenal mucosa of chickens receiving amoxicillin or enrofloxacin. There is evidence that certain antibiotics (including enrofloxacin) may have an adverse effect on humoral defense mechanisms while beneficially affecting cell-mediated immunity (CMI) in chickens [6,7,39]. Madubuike et al. [40] reported that early administration of antibiotics had no effect on humoral immunity in chickens. A study by Wang et al. [41] showed that long-term (42-day) administration of the antibiotics chlortetracycline and salinomycin or a probiotic containing *L. plantarum* strain IMAU10120 to chickens had no effect on the serum concentration of IgY. In the present study, early 5-day administration of enrofloxacin reduced the plasma concentration of IgY, whereas its level was not affected by administration of the probiotic-containing *Enterococcus faecium* and *Bacillus amyloliquefaciens*. Antibiotics directly influence the viability of pathogens and exert an immunomodulatory effect by interacting with the immune system and intestinal epithelial cells (IEC), thereby inhibiting inflammation [42]. 

Research indicates that early administration of probiotics can stimulate humoral immunity in chickens [22,43,44]. In our study, however, early administration of a probiotic containing *Enterococcus faecium* and *Bacillus amyloliquefaciens* to chickens had no effect on the plasma levels of either IgY or IgA. However, it caused a decrease in the level of CRP and ceruloplasmin as well as IL-6 and TNF in the blood. Furthermore, in the group receiving the probiotic, there was a marked increase in the percentage of CD3-Bu-1+ B cells in the blood and spleen of 6-day-old chickens in comparison with the control group. It cannot be ruled out that the period of probiotic administration was too short to improve the humoral immune system response expressed by the level of total IgY and IgA in the blood plasma, but sufficient to lower the level of the acute phase proteins IL-6 and TNF tested. Inflammatory reactions are accompanied by systemic and metabolic changes, together referred to as the acute-phase response (APR). Acute-phase proteins (APP) are proteins whose concentrations in the plasma increase in response to inflammation, infection, or tissue damage. These proteins take part in host adaptation or defence, e.g., C-reactive protein (CRP), or act as transport proteins with antioxidant activity, e.g., ceruloplasmin [45]. In a study by Sohail et al. [46], supplementation of the diet with a probiotic containing *Lactobacillus* *plantarum*, *Lactobacillus acidophilus*, *Lactobacillus bulgaricus*, *Lactobacillus rhamnosus*, *Bifidobacterium bifidum*, *Streptococcus thermophilus*, *Enterococcus faecium*, *Aspergillus oryzae*, and *Candida pintolopesii* reduced the plasma CRP level by 55% in broilers subjected to heat stress. Probiotic bacteria, by fermenting dietary fiber, generate short-chain fatty acids (SCFA) such as acetate, propionate and butyrate, which are absorbed by intestinal cells and used as an energy source for their metabolism [47]. Butyrate has been shown to inhibit NO production, reduce the expression of cytokines such as IL-1β, IL-6, IFN-γ, and IL-10, and inhibit transcription of NF-κB, thereby reducing the production of pro-inflammatory cytokines [48]. In our study, as in the case of early administration of a probiotic, early administration of a therapeutic dose of enrofloxacin reduced the levels of CRP and ceruloplasmin but increased the levels of IL-2 in the plasma of chickens.

## 5. Conclusions

Administration of the antibiotic enrofloxacin or a probiotic containing *E. faecium* and *B. amyloliquefaciens* strains to chickens in their first week of life exerts pronounced immunomodulatory effects on humoral and cellular defense mechanisms in these birds. The changes in the subpopulations of B and T cells immediately following early administration of enrofloxacin or the probiotic were not observed at the age of 35 days. Early administration of enrofloxacin can pose a risk of suppression of humoral immunity, as indicated by the significant decrease in the total IgY concentration in the plasma of the chickens.

## Figures and Tables

**Table 1:** Weight of immune system organs of chickens.

Item	BW kg	Spleen Weight g	Spleen Index ^2^	BF Weight g	BF Index ^2^
6 days of age					
GC ^1^	0.152	0.102 ^b^	0.067	0.249 ^b^	0.163 ^b^
GP	0.156	0.121 ^a^	0.078	0.313 ^a^	0.200 ^a^
GA	0.157	0.121 ^a^	0.078	0.327 ^a^	0.209 ^a^
SEM	0.002	0.003	0.002	0.008	0.006
*p*-value	0.571	0.047	0.066	0.036	0.004
35 days of age					
GC ^1^	2.355	1.993	0.085	4.562	0.194
GP	2.450	2.269	0.092	4.394	0.180
GA	2.345	2.374	0.101	4.397	0.189
SEM	0.035	0.028	0.003	0.075	0.007
*p*-value	0.414	0.068	0.101	0.205	0.696

^a,b^ Means within the same column differ significantly (*p* ≤ 0.05) according to Tukey’s HSD test. ^1^ Treatment: GC received no additive in the drinking water; GP received a probiotic preparation providing *Enterococcus faecium* strain 4a1713 at 1.0 × 10^7^ CFU/L water and *Bacillus amyloliquefaciens* 4b1822 at 1.0 × 10^7^ CFU/L water; GA received 10% enrofloxacin at a dose of 0.5 mL/L drinking water. ^2^ the ratio of spleen or bursa of Fabricius weight to body weight. Abbreviations: BW, body weight; BF, bursa of Fabricius; SEM, standard error of the mean.

**Table 2:** Percentages of T cell and B cell subpopulations in the spleen.

Item	Immunophenotype			
	CD3+CD4+	CD3+CD8a+	CD4+CD8a+	CD3-Bu-1+
6 days of age				
GC ^1^	28.66 ^a^	43.38	3.06	19.54 ^b^
GP	22.55 ^b^	43.42	3.17	25.30 ^a^
GA	19.40 ^b^	40.38	2.79	28.33 ^a^
SEM	1.107	1.042	0.164	1.046
*p*-value	0.001	0.407	0.629	0.001
35 days of age				
GC ^1^	22.09	39.23	1.85	28.46
GP	20.82	45.19	2.00	25.42
GA	21.84	43.26	1.71	28.35
SEM	0.826	1.059	0.090	1.120
*p*-value	0.813	0.059	0.457	0.470

^a,b^ Means within the same column differ significantly (*p* ≤ 0.05) according to Tukey’s HSD test. ^1^ Treatment: GC received no additive in the drinking water; GP received a probiotic preparation providing *Enterococcus faecium* strain 4a1713 at 1.0 × 10^7^ CFU/L water and *Bacillus amyloliquefaciens* 4b1822 at 1.0 × 10^7^ CFU/L water; GA received 10% enrofloxacin at a dose of 0.5 mL/L drinking water. SEM, standard error of the mean.

**Table 3:** Percentages of T cell and B cell subpopulations in the blood.

Item	Immunophenotype			
	CD3+CD4+	CD3+CD8a+	CD4+CD8a+	CD3-Bu-1+
6 days of age				
GC ^1^	13.63	3.12	0.985	5.72 ^b^
GP	14.05	3.28	0.759	7.56 ^a^
GA	12.13	4.08	0.680	7.75 ^a^
SEM	0.977	0.207	0.074	0.306
*p*-value	0.715	0.125	0.219	0.007
35 days of age				
GC ^1^	9.44	6.15	0.687	8.58
GP	8.99	7.83	0.574	8.39
GA	9.36	6.36	0.442	8.45
SEM	0.451	0.356	0.086	0.156
*p*-value	0.915	0.108	0.520	0.879

^a,b^ Means within the same column differ significantly (*p* ≤ 0.05) according to Tukey’s HSD test. ^1^ Treatment: GC received no additive in the drinking water; GP received a probiotic preparation providing *Enterococcus faecium* strain 4a1713 at 1.0 × 10^7^ CFU/L water and *Bacillus amyloliquefaciens* 4b1822 at 1.0 × 10^7^ CFU/L water; GA received 10% enrofloxacin at a dose of 0.5 mL/L drinking water. SEM, standard error of the mean.

**Table 4:** Immune parameters of the blood.

Item	ALB g/L	IgA µg/mL	IgY µg/mL	Cp U/L	IL-2 pg/mL	IL-6 ng/L	TNF ng/L	CRP mg/dL
6 days of age								
GC ^1^	7.99	135.1 ^ab^	1207.5 ^a^	5.23 ^a^	518.9 ^b^	119.3 ^a^	164.3 ^ab^	1.196 ^a^
GP	9.27	111.5 ^b^	1088.8 ^ab^	4.54 ^b^	982.3 ^ab^	92.1 ^b^	132.8 ^b^	1.100 ^b^
GA	8.51	150.7 ^a^	928.3 ^b^	5.37 ^ab^	1163.6 ^a^	123.8 ^a^	189.0 ^a^	1.260 ^a^
SEM	0.264	4.997	40.74	0.161	58.31	4.217	7.361	0.018
*p*-value	0.139	0.002	0.014	0.020	<0.001	0.002	0.004	<0.001
35 days of age								
GC ^1^	17.78	250.2	576.2 ^a^	7.08 ^a^	36.07 ^b^	119.4	215.0 ^ab^	1.210 ^a^
GP	20.88	229.2	501.1 ^ab^	5.82 ^b^	41.21 ^ab^	108.6	180.9 ^b^	1.094 ^b^
GA	18.25	174.8	435.5 ^b^	5.24 ^b^	46.52 ^a^	123.6	235.1 ^a^	1.067 ^b^
SEM	0.762	13.44	15.91	0.202	1.605	4.336	7.247	0.018
*p*-value	0.206	0.056	<0.001	<0.001	0.023	0.356	0.005	<0.001

^a,b^ Means within the same column differ significantly (*p* ≤ 0.05) according to Tukey’s HSD test. ^1^ Treatment: GC received no additive in the drinking water; GP received a probiotic preparation providing *Enterococcus faecium* strain 4a1713 at 1.0 × 10^7^ CFU/L water and *Bacillus amyloliquefaciens* 4b1822 at 1.0 × 10^7^ CFU/L water; GA received 10% enrofloxacin at a dose of 0.5 mL/L drinking water. Abbreviations: SEM, standard error of the mean; ALB, albumin; IgA, immunoglobulin A; IgY, immunoglobulin Y; Cp, ceruloplasmin; IL-2, interleukin 2; IL-6, interleukin 6; TNF-α, tumour necrosis factor; CRP, C reactive protein.

## Data Availability

The data presented in this study are available on request from the corresponding author.
